# Predictive Performance of Trauma Scoring Systems for Mortality in Patients with High-Energy Trauma in the Emergency Department

**DOI:** 10.3390/jcm15155791

**Published:** 2026-07-24

**Authors:** Mehmet Oguzhan Ay, Melih Yüksel, Fatma Betül Çalışkan, Cengizhan Keski, Irem Fidan Yolay, Suna Eraybar, Yeşim Işler, Umut Ocak, Halil Kaya

**Affiliations:** 1 Department of Emergency Medicine, University of Health Sciences, Bursa Yuksek Ihtisas Training and Research Hospital, Bursa 16310, Türkiye; 2 Department of Emergency Medicine, Kestel State Hospital, Bursa 16450, Türkiye; 3 Department of Emergency Medicine, Gemlik State Hospital, Bursa 16600, Türkiye; 4 Department of Emergency Medicine, University of Health Sciences, Bursa City Hospital, Bursa 16110, Türkiye; 5 Department of Emergency Medicine, Medical Faculty, University of Samsun, Samsun 55270, Türkiye

**Keywords:** trauma, Injury Severity Score, Trauma and Injury Severity Score, Rapid Emergency Medicine Score, Shock Index, mortality, emergency department, prognostic models

## Abstract

**Background/Objectives:** Early identification of high-risk trauma patients is essential for optimizing clinical decision-making and improving outcomes. Several trauma scoring systems are available, but their comparative performance for predicting early and short-term mortality remains uncertain. This study compared the prognostic performance of the Injury Severity Score (ISS), Trauma and Injury Severity Score (TRISS), Rapid Emergency Medicine Score (REMS), and Shock Index for predicting both 24-h and 28-day mortality in patients with high-energy trauma. **Methods:** This prospective observational study included 277 consecutive adult patients presenting with high-energy trauma to a tertiary emergency department. ISS, TRISS, REMS, and Shock Index were calculated on admission. The primary outcomes were 24-h and 28-day mortality. Discriminatory performance was assessed using receiver operating characteristic (ROC) analysis. Optimal cut-off values were determined using the Youden index, and pairwise ROC comparisons were performed using the DeLong method. An exploratory multivariable logistic regression analysis was conducted to evaluate factors associated with 28-day mortality. **Results:** The 24-h mortality rate was 1.4% (4/277), whereas the 28-day mortality rate was 5.4% (15/277). For predicting 28-day mortality, TRISS (AUC 0.939, 95% CI 0.898–0.979), REMS (AUC 0.907, 95% CI 0.842–0.971), and ISS (AUC 0.900, 95% CI 0.827–0.973) demonstrated excellent and comparable discrimination, whereas Shock Index showed significantly lower predictive performance (AUC 0.696, 95% CI 0.519–0.874). Pairwise DeLong comparisons demonstrated no significant differences among ISS, TRISS, and REMS, whereas Shock Index performed significantly worse than all three scores. Exploratory multivariable analysis identified antiplatelet therapy, abdominal trauma, head and neck trauma, any comorbidity, and age ≥ 65 years as factors associated with increased odds of 28-day mortality. **Conclusions:** ISS, TRISS, and REMS demonstrated comparable and excellent performance for predicting 28-day mortality following high-energy trauma, whereas Shock Index showed significantly lower discriminatory ability. REMS may provide a practical bedside tool for early risk assessment, while ISS and TRISS remain valuable for comprehensive prognostic evaluation. Because of the limited number of mortality events, the multivariable analysis should be considered exploratory and requires validation in larger prospective multicenter studies.

## 1. Introduction

Trauma remains a major global public health challenge, with injuries and violence accounting for approximately 8% of all deaths worldwide and disproportionately affecting younger populations, particularly in low- and middle-income countries [1,2]. Early identification of patients at risk for clinical deterioration or mortality is a cornerstone of trauma care, as it directly influences clinical decision-making, resource allocation, and patient outcomes [3].

Accurate assessment of injury severity and physiological derangement is therefore essential in the management of trauma patients. Over recent decades, various trauma scoring systems have been developed to facilitate risk stratification by incorporating anatomical, physiological, or combined parameters [4,5]. Among these, the ISS remains one of the most widely used tools for quantifying anatomical injury burden and has been extensively validated across different trauma populations [6].

In contrast, the TRISS integrates anatomical injury severity with physiological parameters and age to estimate survival probability, representing a more comprehensive prognostic model [4]. Simpler bedside tools, such as the REMS, rely on readily available clinical variables and enable rapid risk assessment in time-critical emergency settings [7]. Similarly, the SI, defined as the ratio of heart rate to systolic blood pressure, has been proposed as a practical indicator of hemodynamic instability and early shock in trauma patients [8,9]. Because these scoring systems rely on different anatomical and physiological principles, their prognostic performance may vary according to the timing of outcome assessment and the characteristics of the trauma population.

Despite their widespread use, the predictive performance of trauma scoring systems may vary depending on the study population, clinical setting, and clinical outcomes evaluated [10]. Previous studies have also demonstrated the prognostic utility of individual scoring systems, such as the Rapid Emergency Medicine Score (REMS), in trauma populations [11].

Although the ISS, TRISS, REMS, and SI are among the most frequently used trauma scoring systems, they assess different dimensions of trauma severity. ISS primarily reflects anatomical injury burden, whereas REMS is based on physiological variables that better capture acute clinical deterioration. TRISS integrates anatomical injury severity, physiological status, and age to estimate survival probability, while SI provides a rapid bedside assessment of hemodynamic compromise. Recent systematic reviews, meta-analyses, and comparative studies have demonstrated that no single trauma scoring system consistently outperforms the others across different trauma populations and clinical settings, highlighting the importance of selecting the most appropriate tool according to the clinical objective and timing of assessment [12,13,14,15].

Trauma-related mortality follows a distinct temporal pattern. Deaths occurring within the first 24 h are primarily associated with severe anatomical injury, exsanguination, and irreversible physiological collapse, whereas 28-day mortality additionally reflects the effects of intensive care management, secondary complications, infections, and multiple organ dysfunction. Therefore, evaluating trauma scoring systems using both early and short-term mortality provides a more comprehensive assessment of their prognostic value in patients with high-energy trauma [12,14,15].

Therefore, the present study aimed to compare the prognostic performance of the ISS, TRISS, REMS, and SI for predicting both 24-h and 28-day mortality in patients presenting to the emergency department with high-energy trauma. By evaluating anatomical, physiological, and combined trauma scoring systems within the same cohort, we sought to determine their relative clinical utility for early and short-term risk stratification.

## 2. Materials and Methods

### 2.1. Study Design and Setting

This prospective cohort study was conducted in the Emergency Medicine Department of University of Health Sciences Bursa Yüksek İhtisas Training and Research Hospital, a tertiary care referral center with a high volume of trauma patients. The emergency department serves a large urban population and provides comprehensive emergency and trauma care for patients with both blunt and penetrating injuries. During the study period, consecutive eligible patients presenting with high-energy trauma were prospectively enrolled according to the predefined inclusion and exclusion criteria.

### 2.2. Ethical Approval

Ethical approval for this study was obtained from the Institutional Clinical Research Ethics Committee (Approval No. 2011-KAEK-25/2022-01-21). The study was conducted in accordance with the principles of the Declaration of Helsinki and its subsequent amendments. Written informed consent was obtained from all conscious patients before study enrollment. For patients who were unconscious or otherwise unable to provide informed consent because of the severity of their injuries, written informed consent was obtained from their legally authorized first-degree relatives prior to study participation. Emergency medical treatment was never delayed for study participation. For patients who were unconscious or otherwise unable to provide informed consent, all emergency diagnostic and therapeutic interventions were performed according to standard clinical practice. Written informed consent from the legally authorized first-degree relatives was obtained as soon as feasible after initial stabilization and before study-specific data collection.

All collected data were pseudonymized before statistical analysis and stored in a secure password-protected electronic database accessible only to the study investigators.

### 2.3. Study Population

In this prospective cohort study, 1564 consecutive patients presenting to the emergency department with high-energy trauma between 1 February 2022 and 31 March 2023 were initially screened for eligibility. High-energy trauma was defined based on established mechanisms of injury, including falls from a height of ≥6 m, motor vehicle collisions at speeds ≥65 km/h, vehicle rollovers, ejection from a vehicle, death of another passenger in the same vehicle, pedestrian or cyclist struck at speeds ≥32 km/h, gunshot injuries, explosions, and high-voltage electrical injuries (≥1000 volts).

Patient eligibility was assessed according to the predefined inclusion and exclusion criteria. Only patients with complete data required for calculating all evaluated trauma scoring systems (ISS, TRISS, REMS, and SI) and complete outcome data were included in the final analyses.

### 2.4. Exclusion Criteria

**Figure 1:**
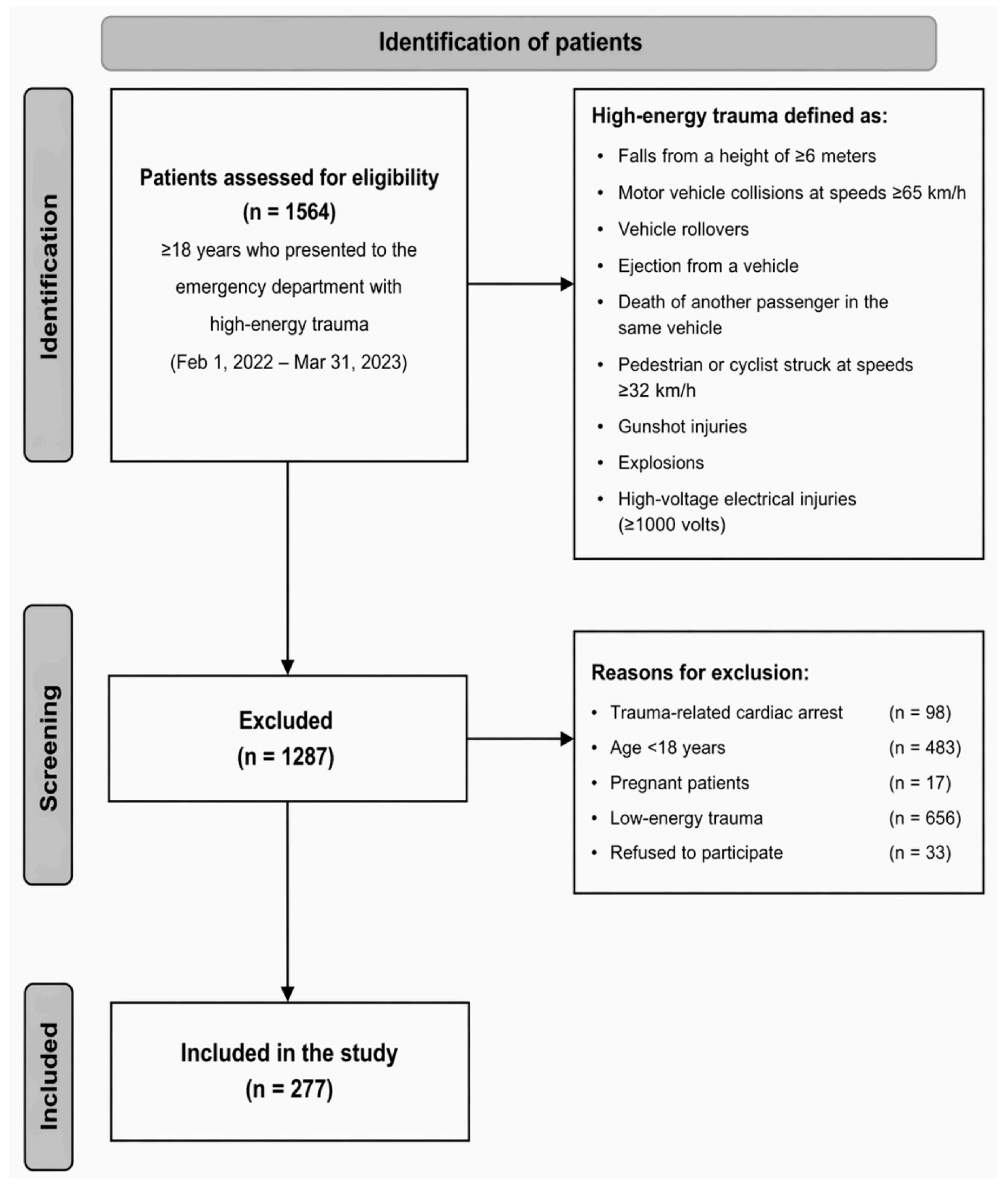
Flow chart of patient enrollment.

Patients were excluded from the study if they experienced trauma-related cardiac arrest before emergency department evaluation, were younger than 18 years of age, were pregnant, had sustained low-energy trauma, or declined to participate in the study. After applying the predefined exclusion criteria, 277 eligible patients were included in the final analysis. The patient selection process is summarized in Figure 1.

### 2.5. Data Collection

Data were collected prospectively using a standardized case report form by the attending emergency physicians at the time of emergency department admission. Recorded variables included demographic characteristics (age, sex), mechanism of injury, vital signs (systolic and diastolic blood pressure, heart rate, respiratory rate, peripheral oxygen saturation, and body temperature), Glasgow Coma Scale (GCS), comorbidities, and medication use.

Clinical outcomes, including hospitalization, intensive care unit (ICU) admission, discharge, and in-hospital mortality, were also documented. Twenty-eight-day mortality was determined using hospital records and follow-up procedures, including review of the hospital information system and telephone contact when necessary. All data were subsequently entered into a secure electronic database for statistical analysis.

Before statistical analysis, data completeness was assessed for all study variables. Missing values were limited to selected laboratory variables, including lactate (122/277, 44.0%), base excess (140/277, 50.5%), INR (34/277, 12.3%), hemoglobin (1/277, 0.4%), platelet count (1/277, 0.4%), mean platelet volume (1/277, 0.4%), neutrophil count (1/277, 0.4%), and ethanol level (258/277, 93.1%). No missing data were present for variables required for calculation of ISS, TRISS, REMS, Shock Index, or for the primary outcome variables. Therefore, no imputation methods were applied. Available-case analysis was performed only for analyses involving laboratory variables and was not used for trauma score calculations, ROC analyses, multivariable logistic regression, or the primary outcome analyses.

### 2.6. Trauma Scoring Systems

For each patient, all trauma scoring systems were calculated using the initial clinical, physiological, and anatomical data obtained at emergency department admission before any definitive therapeutic intervention. All scores were calculated according to their original published methodologies using standardized definitions and variables.

The ISS was calculated based on the Abbreviated Injury Scale (AIS), by squaring and summing the highest AIS scores from the three most severely injured body regions. The Revised Trauma Score (RTS) was calculated using the coded values of the Glasgow Coma Scale (GCS), systolic blood pressure, and respiratory rate.

The TRISS was calculated using the revised TRISS algorithm implemented in MedCalc Statistical Software (version 20.014; MedCalc Software Ltd., Ostend, Belgium). The calculation incorporated ISS, RTS, patient age, and injury mechanism (blunt or penetrating trauma) to estimate each patient’s probability of survival.

Injury severity was coded by emergency physicians experienced in trauma assessment using the Abbreviated Injury Scale definitions before calculation of ISS and TRISS.

The REMS was calculated using age, mean arterial pressure, heart rate, respiratory rate, peripheral oxygen saturation, and GCS score. The SI was calculated as the ratio of heart rate to systolic blood pressure.

All trauma scores were calculated before outcome assessment and were subsequently evaluated for their ability to predict both 24-h and 28-day mortality.

### 2.7. Outcome Measures

The primary outcome measures of the study were 24-h mortality and 28-day mortality. Twenty-four-hour mortality was defined as death from any cause occurring within the first 24 h after emergency department admission, whereas 28-day mortality was defined as all-cause mortality occurring within 28 days of admission.

Twenty-eight-day survival status was determined through review of hospital medical records and the hospital information system. For patients who had been discharged before 28 days, follow-up information was obtained by telephone contact when necessary to ascertain survival status.

### 2.8. Statistical Analysis

All statistical analyses were performed using IBM SPSS Statistics for Windows (version 21.0; IBM Corp., Armonk, NY, USA) and MedCalc Statistical Software (version 20.014; MedCalc Software Ltd., Ostend, Belgium).

Continuous variables were assessed for normality using the Kolmogorov–Smirnov test, and homogeneity of variance was evaluated using Levene’s test. Continuous variables are presented as mean ± standard deviation (SD) or median with interquartile range (IQR), as appropriate according to data distribution. Categorical variables are presented as frequencies and percentages.

Comparisons between groups were performed using the independent-samples *t*-test or the Mann–Whitney U test for continuous variables and the chi-square test or Fisher’s exact test for categorical variables, as appropriate.

Receiver operating characteristic (ROC) curve analysis was performed to evaluate the predictive performance of ISS, TRISS, REMS, and Shock Index for 24-h and 28-day mortality. Discriminatory performance was assessed by calculating the area under the ROC curve (AUC) with 95% confidence intervals (95% CIs). Optimal cut-off values were determined using the Youden index. Pairwise comparisons between correlated ROC curves were performed using the DeLong method.

Because TRISS is expressed as the estimated probability of survival, it was transformed into an estimated probability of mortality (100 − survival probability) before ROC analyses. This transformation allowed all trauma scoring systems to be evaluated using the same clinical outcome (mortality) and did not alter the discriminatory performance of TRISS.

Variables showing statistical significance in univariate analyses and considered clinically relevant were entered into the multivariable logistic regression model to identify factors independently associated with 28-day mortality. Because of the limited number of mortality events, the multivariable analysis should be interpreted as exploratory and hypothesis-generating.

A two-sided *p*-value < 0.05 was considered statistically significant.

## 3. Results

### 3.1. Patient Characteristics

A total of 277 patients with high-energy trauma were included in the final analysis. The median age was 40 years (IQR, 28.5–56.0), and 213 patients (76.9%) were male. Most patients were younger than 65 years (85.6%), while comorbid conditions were present in 50 patients (18.1%) and regular medication use was reported by 37 patients (13.4%).

The most common mechanisms of injury were pedestrian traffic accidents (27.4%), motor vehicle collisions (25.3%), and falls from height (24.2%). Extremity injuries (61.4%) and head and neck injuries (58.5%) were the most frequently affected anatomical regions. During the emergency department stay, 120 patients (43.3%) were admitted to hospital wards, 42 (15.2%) required intensive care unit admission, and 2 patients (0.7%) died in the emergency department. Overall, 24-h mortality was 1.4%, whereas the 28-day mortality rate was 5.4%. Detailed baseline demographic and clinical characteristics are presented in Table 1.

### 3.2. Clinical Findings and Outcomes

At emergency department admission, the median GCS score was 15 (IQR, 15–15), median systolic blood pressure was 125 mmHg (IQR, 110–140), median diastolic blood pressure was 78 mmHg (IQR, 70–84), median heart rate was 85 beats/min (IQR, 76.5–100), median respiratory rate was 15 breaths/min (IQR, 14–16), and median peripheral oxygen saturation was 98% (IQR, 96–99). The mean body temperature was 36.31 ± 0.24 °C.

The median ISS was 16 (IQR, 5–26), the median RTS was 8 (IQR, 8–8), the median TRISS predicted survival probability was 98.8% (IQR, 96.8–99.7), the median REMS was 2 (IQR, 0–4), and the mean SI was 0.85 ± 0.39.

Laboratory findings at admission demonstrated a mean white blood cell count of 11,852 ± 5175/µL, hemoglobin concentration of 13.76 ± 1.92 g/dL, platelet count of 265,020 ± 73,159/µL, mean platelet volume of 10.37 ± 1.10 fL, neutrophil count of 8045 ± 4920/µL, international normalized ratio (INR) of 0.99 ± 0.16, lactate level of 2.67 ± 2.45 mmol/L, and base excess of 1.43 ± 5.81 mmol/L. Detailed clinical and laboratory findings are presented in Table 2.

### 3.3. Comparison of Trauma Scores Between Survivors and Non-Survivors

For 24-h mortality, non-survivors had significantly higher ISS and REMS values and significantly lower TRISS survival probabilities than survivors (all *p* < 0.05). In contrast, SI did not differ significantly between the two groups (Table 3A).

For 28-day mortality, non-survivors likewise demonstrated significantly higher ISS, REMS, and SI values, together with significantly lower TRISS survival probabilities, compared with survivors (all *p* < 0.05) (Table 3B).

Because only four patients died within the first 24 h after admission, the analyses related to 24-h mortality should be considered exploratory and interpreted with caution.

### 3.4. Predictive Performance of Scoring Systems

ROC curve analysis demonstrated excellent discrimination of the ISS for predicting 24-h mortality (AUC: 0.995, 95% CI: 0.986–1.000). The REMS also demonstrated good discriminatory performance (AUC: 0.859, 95% CI: 0.675–1.000), whereas SI showed comparatively limited predictive ability (AUC: 0.644, 95% CI: 0.303–0.986). The TRISS, after transformation to estimated mortality risk, also demonstrated good discriminatory performance.

**Figure 2:**
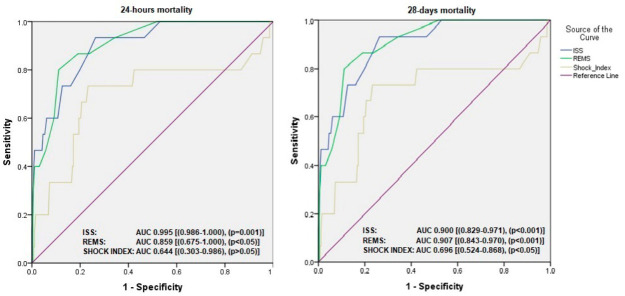
Results of 24-h and 28-day ROC analysis for survival prediction.

For 28-day mortality, TRISS (AUC: 0.939), REMS (AUC: 0.907, 95% CI: 0.843–0.970), and ISS (AUC: 0.900, 95% CI: 0.829–0.971) all demonstrated excellent discriminatory performance, whereas Shock Index showed significantly lower discrimination (AUC: 0.696, 95% CI: 0.524–0.868). Detailed ROC curves are presented in Figure 2.

Formal pairwise comparison of the ROC curves using the DeLong method demonstrated that, for 24-h mortality, the AUC of ISS was significantly greater than that of TRISS (*p* = 0.014), whereas the differences between ISS and REMS (*p* = 0.195) and between ISS and SI (*p* = 0.078) were not statistically significant. REMS demonstrated a significantly greater AUC than SI (*p* = 0.031), while the remaining pairwise comparisons were not statistically significant. Because only four patients died within the first 24 h, these comparisons should be interpreted as exploratory.

For 28-day mortality, no statistically significant differences were observed between ISS and TRISS (*p* = 0.295), ISS and REMS (*p* = 0.901), or TRISS and REMS (*p* = 0.418). In contrast, Shock Index demonstrated significantly lower discriminatory performance than ISS (*p* = 0.028), TRISS (*p* = 0.010), and REMS (*p* = 0.015).

### 3.5. Optimal Cut-Off Values

Optimal cut-off values for each scoring system were determined using the Youden index, and the corresponding sensitivity, specificity, positive predictive value (PPV), and negative predictive value (NPV) are summarized in Table 4.

For 24-h mortality, the optimal cut-off values were ISS ≥ 57, TRISS ≤ 97.0%, REMS ≥ 7, and Shock Index ≥ 1.07. For 28-day mortality, the corresponding optimal cut-off values were ISS ≥ 25, TRISS ≤ 97.0%, REMS ≥ 6, and Shock Index ≥ 0.94.

Across all scoring systems, negative predictive values were consistently high, whereas positive predictive values remained relatively low, reflecting the low mortality rate observed in the study cohort rather than poor discriminatory performance.

### 3.6. Exploratory Multivariable Analysis for 28-Day Mortality

In the exploratory multivariable logistic regression analysis, antiplatelet drug use (OR: 9.09, 95% CI: 2.46–33.60, *p* = 0.001), age ≥ 65 years (OR: 5.03, 95% CI: 1.47–17.15, *p* = 0.010), and abdominal trauma (OR: 7.02, 95% CI: 2.20–22.43, *p* = 0.001) were associated with increased odds of 28-day mortality. Detailed results of the multivariable analysis are presented in Table 5.

This multivariable model should be considered exploratory because of the limited number of mortality events (*n* = 15). The results should therefore be interpreted cautiously and require validation in larger prospective cohorts.

For 24-h mortality, ISS demonstrated an AUC of 0.995 (95% CI: 0.986–1.000), followed by TRISS (AUC: 0.860, 95% CI: 0.758–0.962), REMS (AUC: 0.859, 95% CI: 0.650–1.000), and SI (AUC: 0.644, 95% CI: 0.251–1.000). Because only four deaths occurred within the first 24 h, these estimates and pairwise comparisons should be considered exploratory. In the DeLong analysis, ISS showed a significantly greater AUC than TRISS (*p* = 0.014), while REMS showed a significantly greater AUC than SI (*p* = 0.031). The remaining pairwise differences were not statistically significant.

For 28-day mortality, TRISS demonstrated the numerically highest discriminatory performance (AUC: 0.939, 95% CI: 0.898–0.979), followed by REMS (AUC: 0.907, 95% CI: 0.842–0.971) and ISS (AUC: 0.900, 95% CI: 0.827–0.973). No significant differences were observed among ISS, TRISS, and REMS in pairwise DeLong comparisons (all *p* > 0.05). SI showed lower discrimination (AUC: 0.696, 95% CI: 0.519–0.874) and performed significantly worse than ISS (*p* = 0.028), TRISS (*p* = 0.010), and REMS (*p* = 0.015). The optimal cut-off values determined using the Youden index were ISS ≥ 25, TRISS ≤ 97.0%, REMS ≥ 6, and SI ≥ 0.94.

## 4. Discussion

In this prospective cohort study, we compared the performance of four widely used trauma scoring systems (ISS, TRISS, REMS, and Shock Index) for predicting both 24-h and 28-day mortality in patients with high-energy trauma. Overall, ISS, TRISS, and REMS demonstrated excellent discriminatory performance, whereas Shock Index showed substantially lower predictive ability, particularly for 28-day mortality. Formal pairwise comparisons using the DeLong method further demonstrated that ISS, TRISS, and REMS had comparable discriminatory performance for 28-day mortality, while Shock Index performed significantly less well than each of these scoring systems [16].

Our analysis of 24-h mortality should be considered exploratory because only four early deaths occurred during the study period. Consequently, the exceptionally high AUC observed for ISS should be interpreted with caution, as the limited number of outcome events may have resulted in an overestimation of predictive performance. In addition, although ROC-derived cut-off values demonstrated high negative predictive values, the relatively low positive predictive values primarily reflected the low mortality rate of the study population rather than inadequate discriminatory ability. Therefore, these findings should not be used to guide clinical decision-making until validated in larger prospective multicenter cohorts.

ISS demonstrated excellent discriminatory performance for predicting early mortality, which is biologically plausible because it reflects the anatomical burden of injury, a major determinant of death during the early phase after high-energy trauma. However, formal pairwise ROC comparisons demonstrated a statistically significant difference only between ISS and TRISS, whereas comparisons with REMS and Shock Index were not statistically significant. Furthermore, because only four patients died within the first 24 h, these findings should be interpreted cautiously, as the observed AUC values may overestimate the true predictive performance of ISS. Previous studies similarly support the value of ISS for assessing trauma severity and early mortality, although its dependence on detailed anatomical injury coding may limit its usefulness during the initial emergency department evaluation [17,18,19].

TRISS also demonstrated excellent performance for both early and 28-day mortality. By integrating anatomical injury severity, physiological status, patient age, and injury mechanism, TRISS provides a comprehensive estimate of survival probability and remains one of the most widely accepted prognostic models in trauma research and quality assessment [4,20]. In our study, no statistically significant differences were observed between TRISS, ISS, and REMS for predicting 28-day mortality, suggesting that these three scoring systems provide comparable prognostic performance despite their different methodological approaches. Therefore, the choice among them may depend more on data availability, timing of assessment, and intended clinical application than on meaningful differences in predictive accuracy [21].

REMS likewise demonstrated excellent predictive performance and performed comparably with ISS and TRISS for predicting 28-day mortality. Because REMS relies exclusively on routinely available physiological variables, it can be calculated rapidly at the bedside without requiring anatomical injury coding. This practical advantage makes REMS particularly useful for the initial evaluation and early risk stratification of trauma patients in emergency departments [22,23]. Although REMS is not trauma-specific, its ability to identify physiological deterioration before complete anatomical assessment becomes available may explain its strong prognostic performance. Nevertheless, REMS may also be influenced by pre-existing comorbidities and chronic physiological disturbances; therefore, it should be considered complementary rather than a replacement for trauma-specific scoring systems [23,24].

In contrast, Shock Index demonstrated significantly lower discriminatory performance for predicting 28-day mortality. Pairwise ROC comparisons using the DeLong method confirmed that Shock Index performed significantly worse than ISS, TRISS, and REMS. Although Shock Index is widely recognized as a simple and rapidly obtainable bedside indicator of hemodynamic compromise, it reflects only the relationship between heart rate and systolic blood pressure and therefore captures a limited aspect of the physiological response to trauma [25,26,27].

Our findings are consistent with recent evidence. A large systematic review and meta-analysis by Carsetti et al. concluded that although Shock Index is a valuable bedside indicator, its predictive performance for trauma mortality is only moderate and should be interpreted together with other clinical findings. Similarly, Liao et al. reported that Shock Index is useful for identifying trauma patients at increased risk of adverse outcomes but emphasized that it should be used alongside comprehensive injury-severity assessment [25,26]. Likewise, our recently published study demonstrated that isolated Shock Index showed lower prognostic performance than more comprehensive trauma scoring systems in patients with high-energy trauma [27].

Several factors may explain the relatively modest performance of Shock Index. Younger patients may maintain normal systolic blood pressure despite substantial blood loss through physiological compensation, whereas older patients or those receiving beta-blockers may exhibit blunted heart-rate responses. Consequently, Shock Index may underestimate the severity of circulatory compromise in these populations [28,29]. Nevertheless, because of its simplicity and immediate availability, Shock Index remains useful as an initial screening tool while more comprehensive trauma scoring systems are being completed [29].

The present findings have important clinical implications. Although ISS, TRISS, and REMS demonstrated comparable performance for predicting 28-day mortality, these scoring systems differ substantially in complexity and timing of application. ISS and TRISS are more suitable for comprehensive trauma evaluation and benchmarking, whereas REMS offers the advantage of immediate bedside calculation using routinely available physiological variables [5,11,20,21,22,23,24]. Rather than representing competing approaches, these scoring systems should be viewed as complementary tools that can support different stages of trauma assessment and clinical decision-making.

In the exploratory multivariable analysis, age ≥ 65 years, antiplatelet drug use, and abdominal trauma were associated with increased odds of 28-day mortality. These associations are clinically plausible and consistent with previous evidence indicating that advanced age and frailty are associated with reduced physiological reserve and worse trauma outcomes, while pre-injury antiplatelet or anticoagulant therapy and abdominal trauma may further increase mortality risk [30,31,32,33,34].

However, because only 15 deaths occurred and multiple predictors were included in the regression model, the findings should be interpreted cautiously. The limited number of outcome events may have resulted in model overfitting, and the identified associations should therefore be considered hypothesis-generating rather than confirmatory. Furthermore, because the multivariable model included substantially more candidate predictors than recommended for the observed number of outcome events, the regression analysis was likely underpowered. Consequently, the estimated odds ratios, particularly for variables with very low frequencies such as anticoagulant therapy, should be interpreted with considerable caution and should not be regarded as definitive estimates of independent risk. Validation in larger prospective multicenter cohorts is required before these findings can be incorporated into routine risk-stratification strategies.

## 5. Limitations

This study has several limitations that should be considered when interpreting the findings. First, this was a single-center study conducted at a tertiary trauma center, which may limit the generalizability of the results to other healthcare settings and trauma populations. Second, although the overall sample size was adequate, the number of mortality events was relatively small, particularly for 24-h mortality (*n* = 4). Consequently, analyses related to early mortality, including ROC curve comparisons, should be considered exploratory and interpreted with caution.

Third, the multivariable logistic regression analysis was performed with a limited number of outcome events relative to the number of candidate variables. Although the model identified clinically plausible associations, these findings should be regarded as hypothesis-generating rather than confirmatory and require validation in larger prospective multicenter cohorts.

Fourth, the optimal cut-off values identified using ROC analysis were derived from the present study population and have not been externally validated. Therefore, these thresholds should not be applied routinely in clinical practice without independent validation in different trauma populations. Finally, although ISS, TRISS, REMS, and SI were compared using standardized statistical methods, other trauma scoring systems were not evaluated, and additional prognostic models may provide complementary information in future studies.

Despite these limitations, the prospective design, standardized data collection, comprehensive comparison of four commonly used trauma scoring systems, and formal pairwise ROC comparisons using the DeLong method represent important strengths of the present study and contribute to the robustness of the findings.

## 6. Conclusions

In patients with high-energy trauma, ISS, TRISS, and REMS all demonstrated excellent discriminatory performance for predicting 28-day mortality, whereas SI showed significantly lower predictive accuracy. Pairwise ROC comparisons using the DeLong method demonstrated no significant differences among ISS, TRISS, and REMS, suggesting that these scoring systems provide comparable prognostic performance despite their different methodological approaches.

Among these tools, REMS offers the practical advantage of rapid bedside calculation using routinely available physiological variables, whereas ISS and TRISS provide more comprehensive prognostic assessment once definitive injury evaluation has been completed. Therefore, these scoring systems should be considered complementary rather than competing approaches for trauma risk stratification.

Because the analyses of 24-h mortality and the multivariable logistic regression were limited by the relatively small number of mortality events, these findings should be interpreted cautiously and validated in larger prospective multicenter studies before widespread clinical implementation.

## Figures and Tables

**Table 1:** Baseline Demographic and Clinical Characteristics of the Study Population (*n* = 277).

Variable	Overall (*n* = 277)
**Age**	
Age, years	40 (28.5–56.0)
<65 years	237 (85.6)
≥65 years	40 (14.4)
**Sex**	
Male	213 (76.9)
Female	64 (23.1)
**Nationality**	
Republic of Türkiye	252 (91.0)
Foreign	25 (9.0)
**Comorbidities**	
Any comorbidity	50 (18.1)
Hypertension	26 (9.4)
Coronary artery disease	13 (4.7)
Diabetes mellitus	11 (4.0)
Other comorbidities	21 (7.6)
**Regular medication use**	
Any medication	37 (13.4)
Antihypertensive drugs	18 (6.5)
Antiplatelet drugs	15 (5.4)
Anticoagulants	2 (0.7)
Other medications	18 (6.5)

Data are presented as median (interquartile range) or *n* (%).

**Table 2:** Trauma Characteristics and Clinical Outcomes (*n* = 277).

Variable	Overall (*n* = 277)
**Mechanism of injury**	
Pedestrian traffic accident	76 (27.4)
Motor vehicle collision	70 (25.3)
Fall from height	67 (24.2)
Gunshot injury	20 (7.2)
Electrical injury	2 (0.7)
Other mechanisms	42 (15.2)
**Injured body region** *	
Head and neck	162 (58.5)
Thorax	95 (34.3)
Abdomen	62 (22.4)
Extremity	170 (61.4)
Other regions	42 (15.2)
**Emergency department disposition**	
Discharged	23 (8.3)
Ward admission	120 (43.3)
Intensive care unit admission	42 (15.2)
Transfer to another hospital	57 (20.6)
Refused treatment	23 (8.3)
Emergency department mortality	2 (0.7)
**Mortality**	
24-h mortality	4 (1.4)
28-day mortality	15 (5.4)

Data are presented as *n* (%) unless otherwise indicated. * Patients could sustain injuries involving more than one body region; therefore, percentages do not total 100%.

**Table 3:** (**A**) Comparison of Trauma Scores According to 24-h Mortality. (**B**) Comparison of Trauma Scores According to 28-Day Mortality.

**A.**			
**Variable**	**Survivors (*n* = 273)**	**Non-Survivors (*n* = 4)**	***p*** **-Value**
ISS	16.0 (4.5–26.0)	66.0 (57.0–75.0)	**0.001**
TRISS (%)	98.8 (96.9–99.7)	91.1 (48.4–96.3)	**0.013**
REMS	2.0 (0.0–4.0)	9.0 (3.3–11.0)	**0.010**
Shock Index	0.72 (0.60–0.94)	1.11 (0.64–1.50)	**0.323**
**B.**			
**Variable**	**Survivors (*n* = 262)**	**Non-Survivors (*n* = 15)**	***p*** **-Value**
**ISS**	16.0 (4.0–25.0)	43.0 (27.0–57.0)	**<0.001**
**TRISS (%)**	98.84 (97.42–99.70)	56.7 (35.13–88.2)	**<0.001**
**REMS**	2.0 (0.0–3.0)	7.0 (6.0–11.0)	**<0.001**
**Shock Index**	0.71 (0.60–0.90)	1.13 (0.75–1.62)	**0.011**

Data are presented as median (interquartile range). Comparisons were performed using the Mann–Whitney U test. ISS, Injury Severity Score; TRISS, Trauma and Injury Severity Score; REMS, Rapid Emergency Medicine Score.

**Table 4:** Diagnostic Performance of Trauma Scores for Predicting 24-h and 28-Day Mortality.

**A. Prediction of 24-h Mortality**							
**Score**	**AUC (95% CI)**	Optimal Cut-Off *	**Sensitivity, %**	**Specificity, %**	**PPV, %**	**NPV, %**	***p*** **-Value †**
ISS	0.995 (0.986–1.000)	≥57	100.0	98.5	50.0	100.0	<0.001
TRISS	0.860 (0.758–0.962)	≤97.0%	100.0	74.0	5.3	100.0	<0.001
REMS	0.859 (0.650–1.000)	≥7	75.0	89.0	9.1	99.6	0.001
Shock Index	0.644 (0.251–1.000)	≥1.07	75.0	79.1	5.0	99.5	0.473
**B** **. Prediction of 28-Day Mortality**							
**Score**	**AUC (95% CI)**	Optimal Cut-off *	**Sensitivity, %**	**Specificity, %**	**PPV, %**	**NPV, %**	***p*-Value †**
ISS	0.900 (0.827–0.973)	≥25	93.3	73.7	16.9	99.5	<0.001
TRISS	0.939 (0.898–0.979)	≤97.0%	100.0	77.1	20.0	100.0	<0.001
REMS	0.907 (0.842–0.971)	≥6	80.0	88.9	29.3	98.7	<0.001
Shock Index	0.696 (0.519–0.874)	≥0.94	73.3	76.7	15.3	98.0	0.030
**C. Pairwise Comparison of AUCs Using the DeLong Method**							
**Outcome**		**Comparison**		**Difference in AUC**		***p*** **-Value**	
**24-h mortality**		ISS vs. TRISS		0.135		0.014	
		ISS vs. REMS		0.136		0.195	
		ISS vs. Shock Index		0.350		0.078	
		TRISS vs. REMS		0.001		0.995	
		TRISS vs. Shock Index		0.216		0.328	
		REMS vs. Shock Index		0.215		0.031	
**28-day mortality**		ISS vs. TRISS		−0.039		0.295	
		ISS vs. REMS		−0.007		0.901	
		ISS vs. Shock Index		0.204		0.028	
		TRISS vs. REMS		0.032		0.418	
		TRISS vs. Shock Index		0.242		0.010	
		REMS vs. Shock Index		0.211		0.015	

**AUC**, area under the receiver operating characteristic curve; **CI**, confidence interval; **ISS**, Injury Severity Score; **TRISS**, Trauma and Injury Severity Score; **REMS**, Rapid Emergency Medicine Score; **PPV**, positive predictive value; **NPV**, negative predictive value. * Optimal cut-off values were identified using the Youden index. Higher ISS, REMS, and Shock Index values and lower TRISS survival probabilities indicate increased mortality risk. † The *p*-value tests whether the AUC differs significantly from 0.50. Pairwise ROC curve comparisons were performed using the DeLong method. A negative AUC difference indicates that the second score had a numerically higher AUC.

**Table 5:** Exploratory Multivariable Logistic Regression Analysis for 28-Day Mortality.

Variable	Odds Ratio (OR)	95% Confidence Interval	*p*-Value
Antiplatelet therapy	**9.09**	2.46–33.60	**0.001**
Anticoagulant therapy	**0.05**	0.003–0.86	**0.039**
Abdominal trauma	**7.02**	2.20–22.43	**0.001**
Head and neck trauma	**5.17**	1.18–24.68	0.039
Thoracic trauma	0.44	0.16–1.26	0.127
Extremity trauma	1.36	0.48–3.89	0.566
Any comorbidity	**3.30**	1.12–9.75	**0.031**
Hypertension	0.61	0.13–2.88	0.530
Diabetes mellitus	1.39	0.13–15.16	0.786
Coronary artery disease	**0.15**	0.03–0.68	**0.014**
Age ≥ 65 years	**5.03**	1.47–17.15	**0.010**
Mean arterial pressure	0.99	0.97–1.01	0.457

OR, odds ratio; CI, confidence interval.

## Data Availability

The data supporting the findings of this study are available from the corresponding author upon reasonable request.

## Abbreviations

ISS	Injury Severity Score
TRISS	Trauma and Injury Severity Score
REMS	Rapid Emergency Medicine Score
ROC	Receiver operating characteristic
SI	Shock Index
ICU	Intensive care unit
AIS	Abbreviated Injury Scale
RTS	Revised Trauma Score
GCS	Glasgow Coma Scale
